# Altered Spontaneous Brain Activity in Betel Quid Dependence: A Resting-state Functional Magnetic Resonance Imaging Study

**DOI:** 10.1097/MD.0000000000002638

**Published:** 2016-02-08

**Authors:** Tao Liu, Jian-jun Li, Zhong-yan Zhao, Guo-shuai Yang, Meng-jie Pan, Chang-qing Li, Su-yue Pan, Feng Chen

**Affiliations:** From the Department of Neurology, Nanfang Hospital, Southern Medical University, Guangzhou (TL, GY, SP); Department of Neurology (TL, ZZ); and Department of Radiology, People's Hospital of Hainan Province, Haikou, China (JL, MP, CL, FC) in this site.

## Abstract

It has been suggested by the first voxel-based morphometry investigation that betel quid dependence (BQD) individuals are presented with brain structural changes in previous reports, and there may be a neurobiological basis for BQD individuals related to an increased risk of executive dysfunction and disinhibition, subjected to the reward system, cognitive system, and emotion system. However, the effects of BQD on neural activity remain largely unknown. Individuals with impaired cognitive control of behavior often reveal altered spontaneous cerebral activity in resting-state functional magnetic resonance imaging and those changes are usually earlier than structural alteration.

Here, we examined BQD individuals (n = 33) and age-, sex-, and education-matched healthy control participants (n = 32) in an resting-state functional magnetic resonance imaging study to observe brain function alterations associated with the severity of BQD. Amplitude of low-frequency fluctuation (ALFF) and regional homogeneity (ReHo) values were both evaluated to stand for spontaneous cerebral activity. Gray matter volumes of these participants were also calculated for covariate.

In comparison with healthy controls, BQD individuals demonstrated dramatically decreased ALFF and ReHo values in the prefrontal gurus along with left fusiform, and increased ALFF and ReHo values in the primary motor cortex area, temporal lobe as well as some regions of occipital lobe. The betel quid dependence scores (BQDS) were negatively related to decreased activity in the right anterior cingulate.

The abnormal spontaneous cerebral activity revealed by ALFF and ReHo calculation excluding the structural differences in patients with BQD may help us probe into the neurological pathophysiology underlying BQD-related executive dysfunction and disinhibition. Diminished spontaneous brain activity in the right anterior cingulate cortex may, therefore, represent a biomarker of BQD individuals.

## INTRODUCTION

Betel quid ranks the fourth most frequently consumed psychoactive substance around the globe, following only alcohol, nicotine, and caffeine in prevalence.^1^ There are approximately 600 million BQ consumers, and mostly locates in South Asia, Southeast Asia, and Pacific islands.^2^ Betel quid contains parasympathomimetic properties, which stimulate the muscarinic and nicotinic receptors, thus chewing BQ usually can be a dependency syndrome, with enhanced concentration, mild euphoria, relaxation and postprandial satisfaction, and similarly, it can also have withdrawal syndrome related to sleeplessness, unstable mood, irritability, and anxiety. Moreover, BQ chewing leads to a wide variety of health issues, most notably oral cancer and precancerous conditions, such as leukoplakia and oral submucous fibrosis.^3^ Thus, it is of significance to investigate the neural mechanism underlying the betel quid dependence (BQD).

Assessment of BQ dependence has been generally conducted upon the Diagnostic and Statistical Manual of Mental Disorders-IV (American Psychiatric Association, 2000), the International Classification of Diseases-10 (World Health Organization, 1992) ^4–7^ and some dependence scales for other substances such as opioids^8^ or tobacco.^9^ Recently, Lee^10^ developed an initial instrument specially for measuring BQ dependence: the Betel Quid Dependence Scale (BQDS), which is more suitable for Chinese-speaking chewers and valid for current English-speaking male and female chewers in Guam.^11^

Neuroimaging has been proven a useful tool for investigating neural mechanism of drug dependence. In our previous work, we for the first time demonstrated BQ addiction-associated gray matter volume (GMV) alterations by using voxel-based morphometrey (VBM)-MRI.^12^ The BQD individuals presented decreased GMV in the bilateral dorsolateral prefrontal cortex (dlPFC), anterior cingulate cortex (ACC) and mid-brain. Gray matter volume of these regions also showed negative correlation with the duration of BQD and BQDS.^12^ The findings suggested that dysfunction of the reward system, cognitive system, and emotion system may be a neural basis for BQD individuals.

Morphometric alteration is commonly considered as long-term effect of neural plastics of brain. In contrast, resting-state brain function is a spontaneous and baseline neural activity, which is more feasible to reflect the brain functional response to instantaneous extrinsic behavior, and has biological relationship with brain morphometry. Resting-state functional MR imaging (rs-fMRI) has been regarded as a useful tool to measure spontaneous low frequency brain activity.^13^ Amplitude of low-frequency fluctuation (ALFF)^14^ and regional homogeneity (ReHo)^14^ are 2 usually used parameters for describing regional properties of brain activity in rs-fMRI study. Amplitude of low-frequency fluctuation calculates the neural activity intensity at the single-voxel level,^15^ while ReHo calculates the neural synchronization of a specific voxel with its neighboring ones.^16^ Earlier research demonstrated that ReHo may be more sensitive in respect to discovering regional abnormalities compared with ALFF and that ALFF and ReHo may be complementary to each other in terms of detecting global spontaneous activity.^17^ Hence, more information concerning the pathophysiological framework in the human brain might be obtained by combining ALFF and ReHo together.^17^

In this study, combined ALFF and ReHo were used to detect global spontaneous neural activity in BQD individuals. The relative ALFF and ReHo results were reanalyzed and GMV was voxel-wise regarded as the covariate in order to rule out its effect on the functional results.^18^ Then the interrelations between significantly different brain regions and the BQD severity were detected. Determining such neural mechanism would help design prevention and treatment programs aiming to reduce the prevalence of BQD.

## MATERIALS AND METHODS

### Ethics Statement

This study was approved by the research ethics review board of the People's Hospital of Hainan Province, Haikou, China according to the Declaration of Helsinki (2000). The consent form has been read and signed by each subject before being included in the study.

### Inclusion and Exclusion Criteria

The inclusion and exclusion criteria, as mentioned in our previous work,^12^ are listed concisely in the following. We categorized persons with the use of BQ without tobacco at least once a day for 5 years or more as volunteer candidates for entering BQD group. We administered the BQDS during the first session. High comorbidity of addiction with psychiatric disorders, especially anxiety disorders (generalized anxiety disorder and social anxiety disorder) and affective disorders (including depression), had been shown in cross-sectional studies on samples of patients.^19^ On the screening day, all participants were assessed with the self-rating depression scale (SDS) and self-rating anxiety scale (SAS) to preclude the interference of depression and degrees of anxiety. BQD participants met the criteria for present BQ addiction, as diagnosed by the BQDS > 4,^10^ SDS < 50, and SAS < 50. Persons without use of BQ, areca nut (AN), or tobacco (in all forms) were defined as “control individuals.”

The following participants are excluded: tobacco smokers; persons who use tobacco in different forms without smoke, for example, paan masala and/or gutka; persons with present use of psychotropic drugs; persons with self-claimed systemic diseases, such as epilepsy, neurological disorder, diabetes mellitus, cardiovascular disease, thyroid, and renal disorders; persons with current or past history of any Axis I psychiatric and/or substance use diseases as assessed by a semistructured personal interview; persons who are not able to read and write Chinese; and left handers.

## QUESTIONNAIRE

A questionnaire in simple Chinese acquiring information, including sex and age as well as educational status, monthly income, duration of BQ chewing habit, duration time of quid placement in mouth, and daily dosage of BQ was distributed to all participants. All participants were also assessed for alcohol usage in the past 30 days, including number of drinks per occasion and the average frequency of drinking because wine has played an important role in Chinese social aspects of life.

### Study Participants

At first, 38 BQD volunteers and 36 control individuals recruited from a residential area of Wanning City of Hainan province underwent MRI scanning, but only 33 BQD volunteers and 32 control individuals were included because of arachnoid cyst, angiocavernoma, lacunar infarction, and excessive head movement, respectively.

### Magnetic Resonance Imaging Data Acquisition

Magnetic resonance imaging data were obtained on a Siemens Verio3T MRI scanner using a standard 6-channel head coil (Erlangen, Germany) in the Department of Radiology, People's Hospital of Hainan Province from March 2013 to September 2014. All BQ users should not chew BQ on the scanning day. During scanning, the subjects were required to stay awake with their eyes closed and their heads still, thinking of nothing in particular. In order to rule out gross cerebral pathology, a routine structure MR scan was conducted. Anatomical images of the functional slice locations were collected with spin-echo imaging in the axial plan parallel to the Anterior Commissure-Posterior Commissure line. Whole-brain functional images were obtained with a T2∗-weighted echo planar image sequence sensitive to blood oxygen level dependent contrast (repetition time = 2000 milliseconds, echo time = 30 milliseconds, field of view = 240 × 240 mm^2^, flip angle = 80°, image matrix = 64 × 64, voxel size = 3.75 × 3.75 × 5 mm^3^; each brain volume included 31 axial slices; each functional run comprised 240 volumes). Gray matter (GM) volumes of these participants were also calculated for covariate. A high-resolution T1-weighted structural image was obtained using a magnetization-prepared rapid gradient-echo sequence, the parameters were described in great detail in our VBM investigation report.^12^

### Magnetic Resonance Imaging Data Postprocessing

Structural MRI studies have indicated that addiction patients reveal GM loss in various brain regions.^20^ The loss may have local effects on functional images and therefore become a possible confounding factor during the change assessment of cerebral function in addiction patients. To solve this problem, a VBM analysis was first carried out. The VBM8 tool-box (http://dbm.neuro.uni-jena.de/vbm) with default parameters running in the statistical parametric mapping-8 (SPM8, http://www.fil.ion.ucl.ac.uk/spm) software was used to conduct structural analysis. In short, after head-motion correction, individual T1-weighted anatomical images were coregistered to the mean functional images with a linear transformation.^21^ By means of the unified segmentation algorithm, the transformed structural images were then segmented into white matter, gray matter, and cerebrospinal fluid and normalized spatially in the Montreal Neurological Institute (MNI) space.^22^ The segmentation algorithm is deemed to have the capability of addressing the circularity problems of registration and tissue classification in optimized VBM. Next, the GM maps were coordinated to make up for the effect of the spatial normalization and smoothed using a 4-mm Gaussian kernel. The resultant maps might apply to statistical analysis.

We preprocessed the fMRI imaging data with the toolbox Data Processing Assistant for Resting-State functional MR imaging (DPARSF; http://www.restfmri.net/forum/DPARSF)^23^ through an rs-fMRI data analysis toolkit (REST1.8; http://www.restfmri.net) and SPM8 (http://www.fil.ion.ucl.ac.uk/spm/), removing the first 10 volumes of each functional time series for the magnetization equilibrium. Slice timing and realignment were carried out for head motion correction. Spatial normalization to the standard MNI echo-planar imaging template was utilized in the Statistical Parametric Mapping package. Then the functional images were spatially normalized to standard coordinates and resampled to 3 × 3 × 3 mm^3^.^22^ All participants with head motion >1.5° rotation in any directions or >1.5 mm translation were ruled out. No statistical distinction was found between 2 groups for 6 head motion parameters (2-sample *t* test, all *P* > 0.05). Lastly, white matter signal, global signal,^24^ cerebral-spinal fluid signal and 6 parameters coming from head motion correction were excluded as “nuisance regressors” by linear regression.

### Amplitude of Low-frequency Fluctuation and Regional Homogeneity Analyses

Amplitude of low-frequency fluctuation and ReHo analyses were performed using REST software. For ALFF analysis, the resampled images were first smoothed with a Gaussian kernel of 4 mm before linear trend and band-pass filtering (0.01–0.08 Hz) were carried out to remove the effects of high-frequency noise and low-frequency drift. Next, the time series were transformed to the frequency domain with a fast-Fourier transform. Via calculating and averaging the square root of the power spectrum across 0.01–0.08 Hz within each voxel, the raw ALFF value was obtained, which was extracted and averaged to calculate the global mean ALFF value afterwards. Finally, the raw ALFF values of all voxels were divided by the global mean ALFF values for standardization. The resulting ALFF value in a specific voxel reflected the degree of raw ALFF value relative to the average ALFF value of the entire brain.^25^

Regional homogeneity analysis on preprocessed images was also performed. After linear trend and band-pass filtering were carried out, we obtained ReHo images by calculating the concordance of the Kendall coefficient of the time series of a specific voxel with its 26 nearest neighbors.^16^ Then the ReHo value of each voxel was standardized via dividing the raw value by the global mean value, which was achieved using the same method when the global mean ALFF value was determined. Eventually, we smoothed the data with a Gaussian kernel of 4 mm in order to statistically analyze further.

### Statistical Analysis

Using Statistical Product and Service Solutions (SPSS) software (version 16.0; SPSS, Inc, Chicago, IL), we compared the demographic and clinical variables between the BQD group and the control group. An independent 2-sample *t* test was adopted for continuous variables, and a χ^2^ test performed for proportions. *P* values less than 0.01 were regarded as statistically significant.

To study the inter-group differences of ALFF and ReHo values, 2-sample *t* tests were carried out using the REST software (within a brain mask). To exclude GM volume effect on the functional results, the relative ALFF and ReHo results (2-sample *t* tests) were reanalyzed, with GMV as the covariate in a voxel-wise manner.^18^ Age, sex, as well as education levels were also imported as covariates. Group differences were corrected by means of Monte Carlo simulation combining a height threshold of *P* < 0.01 with cluster *P* < 0.05 to result in a family-wise error rate of 5 percent. Therefore, a corrected *P* < 0.01 was achieved using a minimum cluster size of 20 voxels according to different smoothing kernel of the statistical maps (AlphaSim correction).

### Correlation Analysis

Cerebral regions indicating obvious ALFF and ReHo alterations between the patients with BQD and healthy controls were regarded as the regions of interest (ROIs). We averaged the ALFF and ReHo values of the voxels in the ROIs in order to present the ALFF and ReHo of the ROIs. We then performed correlation analysis of Pearson or Spearman between the ALFF, the ReHo values of the ROIs and the BQDS, duration, SDS, and SAS of the patients for the purpose of evaluating their relation according to the normal test results. Next, to recognize the relation between local ALFF and ReHo abnormalities in rACC, we carried out a bivariate correlation between the 2 measurements. In short, the average ALFF and ReHo values of rACC with significant differences were extracted on an individual basis and then correlated with one another.

## RESULTS

### Demographics and Clinical Characteristics

65 subjects (33 BQD users and 32 controls) were included in final data analysis. Individuals in the BQD group and control-group were not different in sex and age. Although the SDS scores in BQD group were remarkably lower than those in controls, the results of emotional status assessed by SAS and SDS failed to reach cut-off for clinical significance on average. None of the participants is alcoholic. Alcohol consumptions in the past 30 days in BQD and health control (HC) group were 200.2 ± 34.8 g and 189.0 ± 33.4 g, respectively. Therefore, we concluded that there is no alcohol-caused effect on BQD usage. Most participants were rubber tappers in state farms, having a superior cultural and economic status than volunteers in other addiction studies. No significant differences were observed in monthly income, education levels, and alcohol consumption in the last 30 days between the 2 groups (*P* values >0.05). Participants reported that they had been chewing BQ with dependency syndrome for a mean length of 20.6 ± 6.9 years (range 7–31 years), a mean BQDS of 10 ± 3.4 (range 5–16), and averagely consumed 342 ± 106 g/day BQ (range 200–500 g/day) daily. Betel quid chewers placed BQ in their mouth and did not spit out the remnants until an average of 7.6 ± 2.4 minutes (range 3 to 12) passed. The demographics of BQD and healthy control participants were outlined in Table 1.

**TABLE 1:**
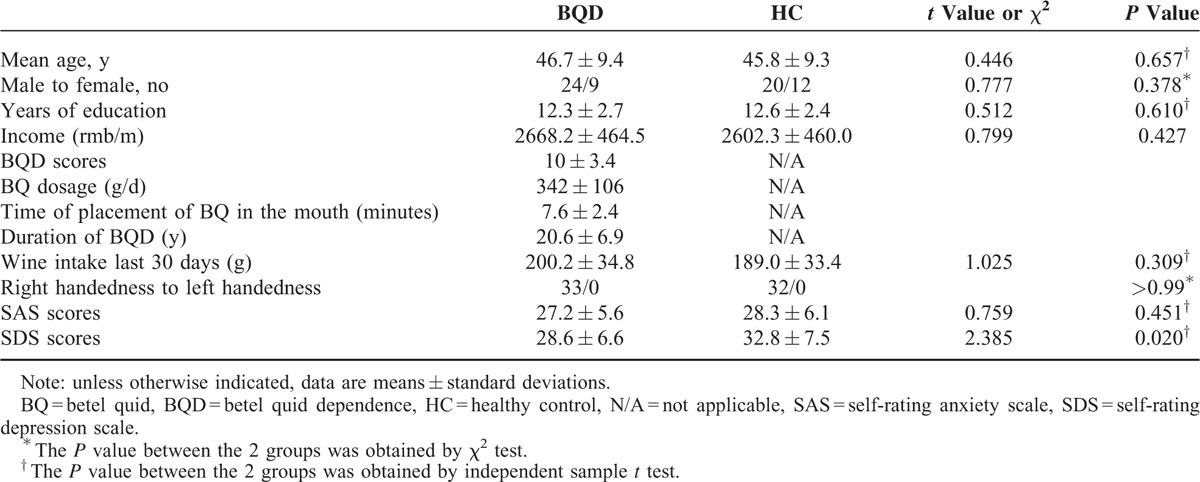
Demographics and Clinical Characteristics of Participants

### Amplitude of Low-frequency Fluctuation and Regional Homogeneity Results

**FIGURE 1:**
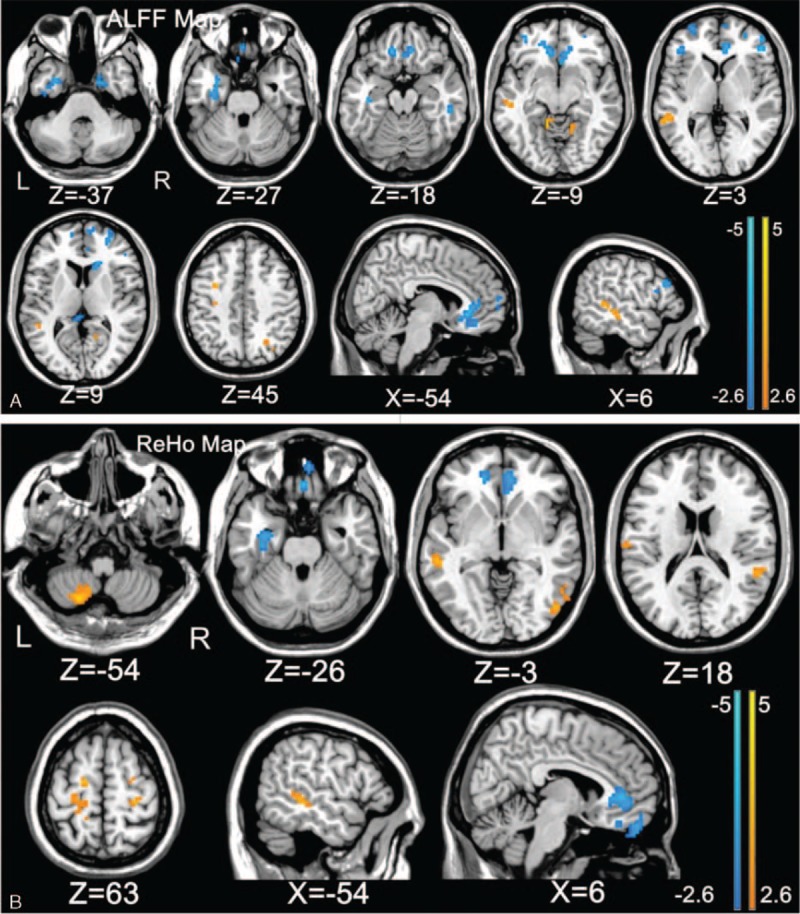
ALFF (A) and ReHo (B) differences between BQD individuals and healthy control subjects (*P* < 0.01, AlphaSim corrected). A, Compared with healthy subjects, patients with BQD showed significantly decreased ALFF in left fusiform, medial prefrontal cortex; right parahippocampal, anterior cingulate cortex; bilateral dorsolateral prefrontal cortex, and increased ALFF in the left precentral, superior temporal gyrus, paracentral lobule; and right calcarine, bilateral lingual. B, Compared with healthy subjects, patients with BQD showed significantly decreased ReHo in the left fusiform, right rectus, and bilateral anterior cingulate cortex and increased ReHo in the left cerebellum posterior lobe, middle temporal gyrus, precentral, paracentral lobule and right superior temporal gyrus, inferior temporal gyrus, inferior occipital gyrus, precentral. For display purposes only, all statistical maps are overlaid on a T1-weighted Montreal Neurological Institute template using MRIcron. ALFF, amplitude of low-frequency fluctuation, BQD = betel quid dependence, ReHo = regional homogeneity.

Gray matter volumes of these participants were also calculated for covariate (see details in our previous report).^12^ Significant differences in spontaneous cerebral activity were found between the 2 groups. In BQD group, the ALFF and ReHo values significantly decreased in the prefrontal cortex (bilateral ACC, bilateral dlPFC), left medial prefrontal cortex (mPFC), right rectus, and some temporal regions (left fusiform and right paraHippocampal) (Figure 1, Table 2).

**TABLE 2:**
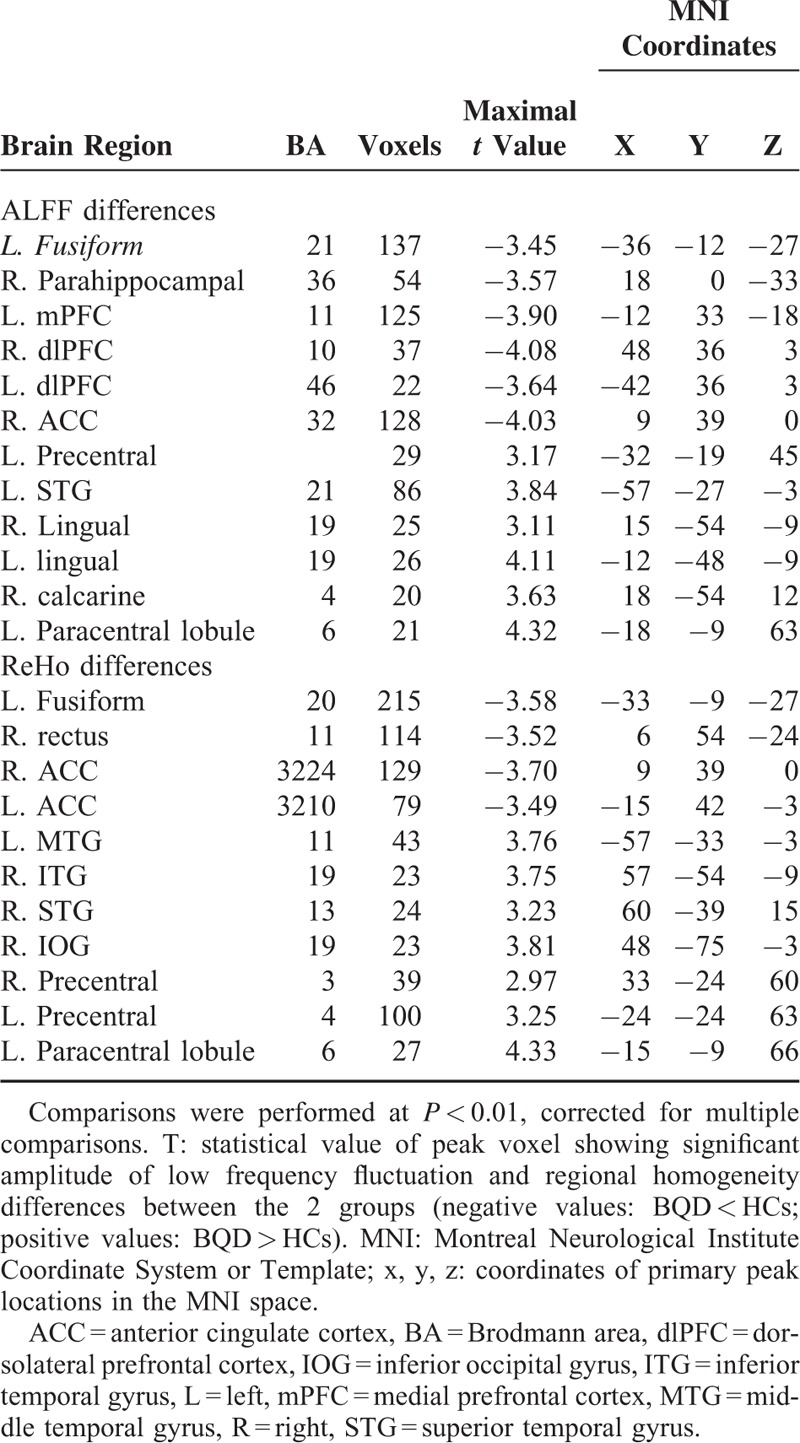
Differences in Amplitude of Low-frequency Fluctuation and Regional Homogeneity Values Between the Betel Quid Dependence Patients and Control-group Members (*P* < 0.01, AlphaSim Corrected)

Amplitude of low-frequency fluctuation and ReHo values in the primary motor cortex area (M1) (bilateral precentral, left paracentral lobule) remained higher in patients with BQD than in healthy controls (Figure 1, Table 2). Amplitude of low-frequency fluctuation and ReHo values also increased in the temporal lobe (left middle temporal gyrus [MTG], right inferior temporal gyrus (ITG), bilateral STG), occipital lobe (right inferior occipital gyrus [ICG], right calcarine, bilateral lingual) respectively (Figure 1, Table 2). The ALFF values increased significantly in the left cerebellum posterior lobe (CPL) as well (Figure 1B, Table 2).

### Correlation Analysis

**FIGURE 2:**
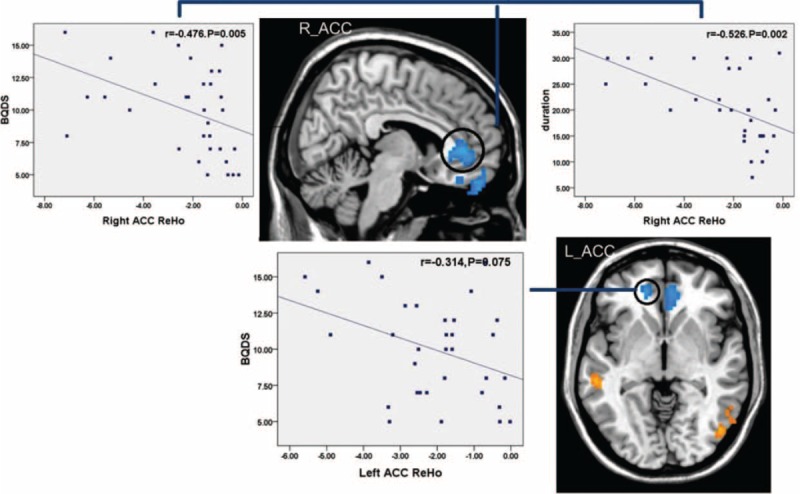
Correlation results between regional homogeneity alteration and duration and BQDS. Spearman correlation reveals that regional homogeneity of the right anterior cingulate cortex showed a negative correlation with BQD score and duration of BQD (*r*1 = −0.476, *P*1 = 0.005 and *r*2 = −0.526, *P*2 = 0.002, respectively), left anterior cingulate cortex showed a negatively correlation trend with BQD scores (*r*3 = −0.314, *P*3 = 0.075) in BQD individuals. BQD = betel quid dependence.

**FIGURE 3:**
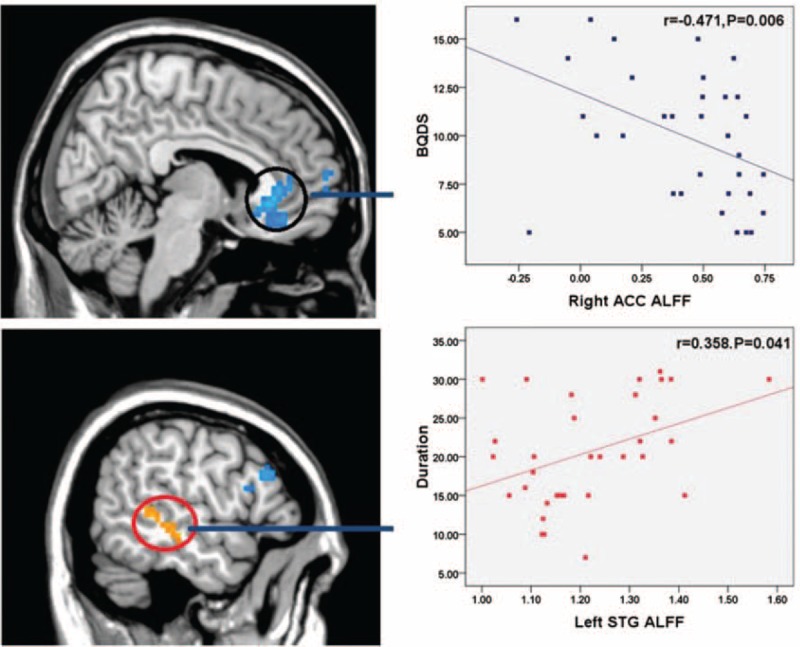
Correlation results between amplitude of low-frequency fluctuation alteration and duration and BQD score. Spearman correlation analyses reveals that amplitude of low-frequency fluctuation of the right anterior cingulate cortex showed a negative correlation with BQD score (*r*4 = −0.471, *P*4 = 0.006), left superior temporal gyrus showed a positively correlation with duration (*r*5 = 0.358, *P*5 = 0.041) in BQD individuals. BQD = betel quid dependence.

As shown in Figures 2 and 3, Spearman correlation analyses revealed that ReHo value of the rACC displayed a negative correlation with BQDS and duration of BQD (*r* = −0.476, *P* = 0.005 and *r* = −0.526, *P* = 0.002, respectively), left ACC showed a negative correlation trend with BQDS (*r* = −0.314, *P* = 0.075) in BQD individuals. Amplitude of low-frequency fluctuation value of the rACC showed a negative correlation with BQDS (*r* = −0.471, *P* = 0.006), left STG showed a positive correlation with duration (*r* = 0.358, *P* = 0.041) in BQD individuals. Our results showed no correlation between SAS and neural activity changes. There was no correlation between SDS and neural activity changes too although the SDS scores in BQD group had a downward trend.

**FIGURE 4:**
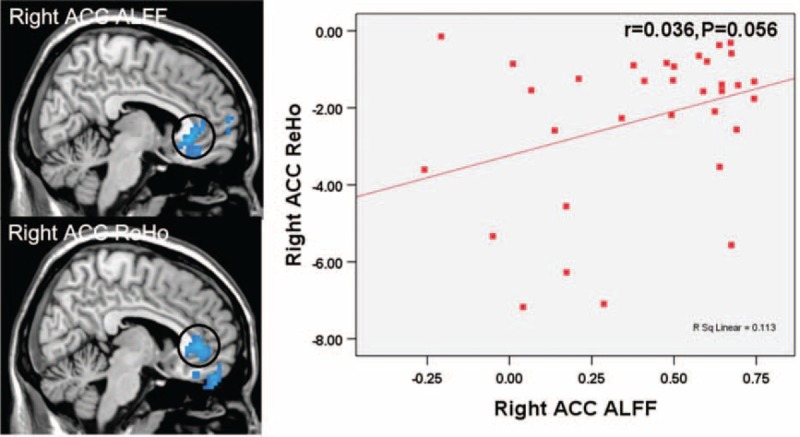
Correlations between the amplitude of low-frequency fluctuation and regional homogeneity values extracted from significantly different regions in right anterior cingulate cortex (bivariate correlation). The mean amplitude of low-frequency fluctuation values had a slightly positive correlation with the mean regional homogeneity values in right anterior cingulate cortex (*r*6 = 0.336, *P*6 = 0.056).

The bivariate correlation analyses revealed that the ALFF and ReHo values extracted from the rACC were significantly correlated with each other (*r* = 0.336, *P* = 0.056). (Figure 4)

## DISCUSSION

In the current rs-fMRI study, we investigated the potential alterations of brain functions associated with duration and severity of BQD. Interestingly, we found the SDS scores in BQD group were significant lower than those in controls in comparison of clinical characteristics although which failed to reach cut-off for clinical significance on average. It has been reported that the extract of AN has potential for the treatment of depression,^1^ so the SDS scores difference may be implicated in those antidepression effect. These, of course, need further researches.

As far as we know, the present study represents the first attempt to determine whether and where spontaneous brain activity abnormalities in BDQ. Amplitude of low-frequency fluctuation and ReHo analyses, which are based on diverse neurophysiology mechanisms, have been performed to investigate the intrinsic neuropathology of different mental diseases.^26,27^ Amplitude of low-frequency fluctuation analysis demonstrates neural intensity^28^ while ReHo analysis indicates neural coherence.^16^ The coexisting functional intensity and coherence abnormalities in such regions may indicate greater severe functional alterations compared with those shown by a single analysis. Would tell from the technology, GMV of these participants were also a possible confounding factor, so VBM-investigation of these participants were also calculated for covariate to rule out its effect on assessment of cerebral function. In this rs-fMRI study we found the more changed brain areas in prefrontal cortex (PFC), some temporal lobe, M1, the occipital lobe and the left CPL compared with the VBM-investigation,^12^ also some alterations in the VBM-investigation such as midbrain, right hippocampal and right precuneus did not appeared in this rs-fMRI study. Hence, we believe our findings are reassuring in terms of comprehending executive dysfunction and disinhibition in BQD individuals.

The mPFC activation, as has been demonstrated, might integrate value across different stimulus dimensions or different stimuli.^29^ Previous fMRI studies indicated that reduced neural activity in the prefrontal gurus was related to maladaptive decision-making^30^ and cognitive control,^31^ respectively. While neurophysiological studies suggest that neurons encoding reward has been reported in the orbital frontal gyrus (OFC) as well as the dlPFC,^32^ these outcomes are in accordance with reward information entering the PFC by way of the OFC, where it is passed to the dlPFC and used for controlling behavior.^33^ Based on the previous studies and our results, we speculated that the decreased ALFF or ReHo values in PFC (bilateral dlPFC, left mPFC and right rectus [belong to OFC]) manifested decision-making and goal driven behavior abnormalities caused by BQD.

It was found in the correlation analysis that the decreased spontaneous brain activity in the right rostral ACC using ALFF and ReHo methods were negatively correlated with BQDS in the BQD group, which are also demonstrated in right rostral ACC by ALFF and ReHo bivariate correlation analyses. The role of the ACC has led to its inclusion in many major theories of addiction, where it is believed to form part of an inhibitory system that exercises control over reward-related behavior.^34,35^ Studies of heroin and cocaine dependent individuals have shown that hypoactivation in the rostral ACC is associated with deficits in response inhibition and impulse control.^36,37^ In addition, the model of addiction proposed by Volkow et al^38^ also confirmed the influence of the ACC's dysfunction upon the addicts.^39^ Our outcomes of the decreased ALFF and ReHo in right rostral ACC negatively correlated with BQDS, may at least in some degree, be in accordance with cognitive management and behavioral dysfunctions directed by goal in BQ addiction. In turn, it also suggests that the spontaneous brain activity in right rostral ACC can act as a reliable biomarker in the exploration of cerebral function on BQD.

In addition, the ReHo values in right rostral ACC were also in negative correlation with duration of BQ use. The result indicates the cumulative effect of BQ consumption, that is, the duration of BQ use negatively correlates with the mean ReHo value in these regions. Also, past voxel-based morphometric studies have demonstrated this effect in heroin use.^40^ Combining previous studies, we argued that BQD can also cause damage to the brain function and its structure, progressively.^12^ Therefore, early intervention plays an important role in BQD treatment.

The increased spontaneous brain activity regions mainly distributed in the M1, temporal lobe, and some regions of occipital lobe in BQD individuals. Mehta^41^ reported that M1 and primary sensory cortex (S1), supplementary motor area, and cerebellum were active during pedaling, and the intensity of activity in these areas increased with increasing pedaling rate and complexity. At least in some degree, the ALFF and ReHo in M1 increased probably because most of our BQD individuals are rubber tappers who have long been engaged in the tapping. The study selects the right-handed subjects so that the activation range and extent in the left M1 area are obvious than the right side. Drug cue-elicited activity in the occipital cortex is commonly demonstrated and has been found in previous meta-analyses of drug cue reactivity.^42–44^ More recently, numerous investigations have described significant drug cue-elicited activity in visual cortex that directly relates to a host of clinical factors,^45–47^ which is compatible with emerging literature regarding the role for primary visual cortex in reward processing.^48,49^ Occipital activation (right ICG, right calcarine, and bilateral lingual) may be associated with chewing visual memory or other complex visual information on the integration of the relevant BQ behaviour.

The temporal lobe has been involved in processing visual information related to emotional content, and the memory trace can be gradually consolidated over time in the forming process of long-time memory.^50^ Medial temporal lobe structures that are critical for long-time memory include the hippocampus, along with the surrounding hippocampal region consisting of the perirhinal, parahippocampal, and entorhinal neocortical regions.^50^ The primary function of the ITG is associated with visual stimuli processing, namely visual object recognition.^50^ The MTG has been connected with processes as different as contemplating distance, recognition of known faces, and accessing word meaning while reading.^50^ The STG has been involved in the perception of emotions in facial stimuli.^50^ The fusiform gyrus has been linked with various neural pathways related to recognition.^50^ What is more, in most functional neuro-imaging studies,^51–53^ diverse findings have been reported and it has been indicated that the activated MTG and fusiform gyrus were associated with the game urge/craving,^54^ semantic processing,^55^ disembodiment,^52^ and working memory^51–53^ in internet gaming disorder. Besides, a number of previous cue-reactivity studies focusing on adolescents with alcohol use disorder,^56,57^ cannabis^58^ and gambling disorder^59^ had reported similar findings (namely dysfunction in the fusiform and MTG). It is believed to manifest the subject's avoidance behavior for clues to stimulate or participate in the reward system. Taken together, we speculated that BQD would impair temporal information processing in retention of visual memories and emotion association, and the increased activity in temporal regions (left MTG, right ITG, and bilateral STG) could act as positive reinforcement factor to make chewers to be addicted to BQ chewing, which was in accordance with that the duration of BQD was positively correlated with ALFF value in the left STG. It is also noteworthy that the reduced activity of the left fusiform gyrus and the right paraHippocampal in BQ addicts was detected in this current study. Based on existing literature, these findings were hard to be interpreted as the reflection of drug use-related changes of brain regions at present. Nevertheless, they provided new clues for exploring the deep mystery of human brain and could stimulate more future investigations.

We also found increased ALFF values in the left CPL in BDQ group. It was observed in previous PET and fMRI studies that drug-conditioned cue elicited the increased metabolism and activation of cerebellum.^60,61^ Glucose metabolism was greatly increased in the cerebellum when addicts performed reward expectation tasks,^62^ which suggested that the cerebellum is also included in drug-conditioned memories in addicts. And the cerebellum has been noted and described to play a compensatory role in inhibitory control ^63^ and decision-making behavior in addicts.^64^ Based on the previous studies and our results above, we argue that the altered cerebral function in left CPL might reflect the neuroadaptation and reorganization of cerebellar functional network caused by BQD. These, of course, need to be confirmed by further researches.

As mentioned in our previous work,^12^ the similar limitations of this study are worth pointing out. First, as the research is cross-sectional, we can only study the abnormal ReHo or ALFF in the BQD, not able to definitely assert the causation of the BQD and the ReHo or ALFF abnormalities because certain subjects have abnormal features in some of their cerebral areas and thus are relatively prone to have BQ addiction development with this pre-existing condition. Therefore, a blanket experimental design is necessary to further consider the cause and effect issues in future work. Nonetheless, we recommended that the present research results be considered as the outcome of BQ dependence. Second, no diagnostic criterion for BQD-associated executive dysfunction or disinhibition existed, and our comprehension of the outcomes was restricted by the absence of objective and particular neurocognitive evaluation. Researchers are incapable of exploring brain patterns of BQD individuals until clinical assessment becomes available. Third, considering the research suggesting that ACC abnormalities involve in addiction more generally, that reduced right ACC spontaneous brain activity would also incur risk for other aspects of problematic substance use is likely, and our findings need further verification with other drug dependence. Lastly, cerebral arterial stenosis or carotid artery stenosis, which can potentially affect cerebral blood flow and cerebral blood volume, had not been excluded using ultrasound or MRA.

In conclusion, the prominent alteration of spontaneous neural activity in BQD was found mainly in brain areas related to PFC, fusiform, M1, temporal lobe, and some regions of occipital lobe, which might reflect the neural plasticity of cerebral functional network caused by BQD. Early intervention may be a valuable therapeutic strategy in BQD treatment.
